# Supplementary figures and images for: Relationship between no-visitation policy and the development of delirium in patients admitted to the intensive care unit
Source: PLoS One. 2022 Mar 9;17(3):e0265082. doi: 10.1371/journal.pone.0265082 (PMC8906646; doi:10.1371/journal.pone.0265082)

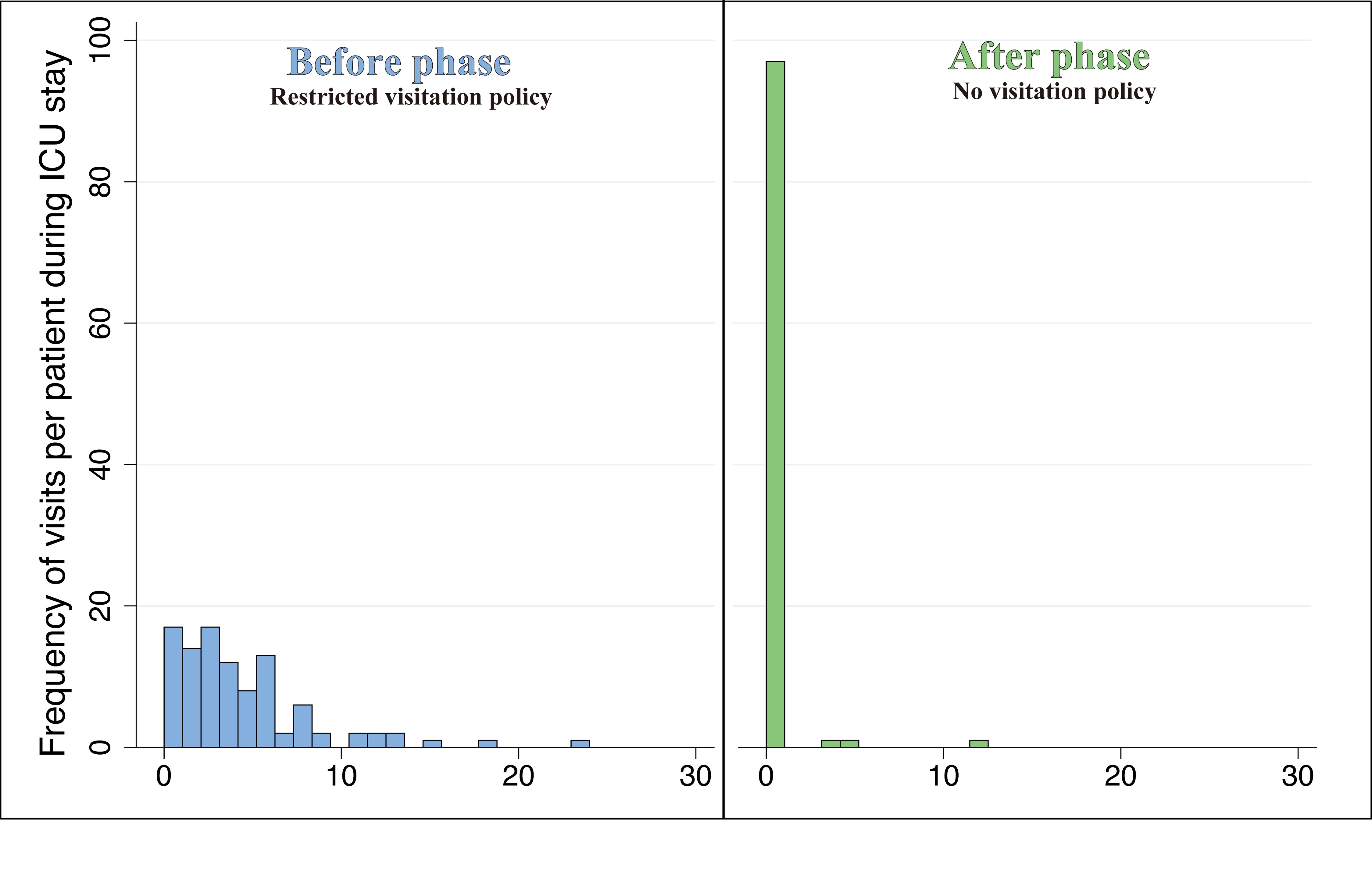

Supplement: S1 Fig — (TIF) [file pone.0265082.s001.tif]
