# Supplementary material for: Relationship between no-visitation policy and the development of delirium in patients admitted to the intensive care unit
Source: PLoS One. 2022 Mar 9;17(3):e0265082. doi: 10.1371/journal.pone.0265082 (PMC8906646; doi:10.1371/journal.pone.0265082)
Supplement: S1 Table — (DOCX) [file pone.0265082.s002.docx]

**S1 Table. Estimates of the adjusted odds ratios of variables on the incidence of delirium in the multivariate logistic regression.**

| **Variable** | **Adjusted**  **odds ratio** | **95% CI** | **p-value** |
| --- | --- | --- | --- |
| **No-visitation policy** | 0.714 | 0.354-1.415 | .339 |
| **Age** | 0.989 | 0.964-1.015 | .416 |
| **Male** | 1.414 | 0.701-2.885 | .336 |
| **Dementia** | 5.904 | 1.788-24.175 | .007 |
| **Emergency surgery** | 1.737 | 0.509-7.038 | .400 |
| **APACHE II**^a^ | 1.109 | 1.055-1.170 | <.001 |
| **Benzodiazepine**  **use** | 2.599 | 0.596-18.299 | .252 |
| **Mechanical ventilation use** | 3.446 | 1.716-7.062 | .001 |

^a^APACHEⅡ score was calculated without age related score
