# Supplementary material for: Relationship between no-visitation policy and the development of delirium in patients admitted to the intensive care unit
Source: PLoS One. 2022 Mar 9;17(3):e0265082. doi: 10.1371/journal.pone.0265082 (PMC8906646; doi:10.1371/journal.pone.0265082)
Supplement: S2 Table — (DOCX) [file pone.0265082.s003.docx]

**S2 Table. Estimates of the adjusted hazard ratios of variables on the development of delirium in the Cox proportional-hazards models.**

To clarify the relationships between the duration of mechanical ventilation and the incidence of delirium, this analysis substituted one of the covariates in the main analysis, mechanical ventilation use, for duration of mechanical ventilation.

| **Variable** | **Adjusted**  **hazard ratio** | **95% CI** | **p-value** |
| --- | --- | --- | --- |
| **No-visitation policy** | 0.938 | 0.644-1.367 | .741 |
| **Age** | 1.000 | 0.986-1.014 | .970 |
| **Male** | 1.120 | 0.753-1.667 | .573 |
| **Dementia** | 2.034 | 1.224-3.382 | .006 |
| **Emergency surgery** | 1.791 | 0.998-3.215 | .050 |
| **APACHE II**^a^ | 1.044 | 1.019-1.070 | <.001 |
| **Benzodiazepine**  **use** | 1.040 | 0.550-1.965 | .902 |
| **Duration**  **of mechanical ventilation (days)** | 1.030 | 0.989-1.074 | .149 |

^a^APACHEⅡ score was calculated without age related score
