# Supplementary material for: Relationship between no-visitation policy and the development of delirium in patients admitted to the intensive care unit
Source: PLoS One. 2022 Mar 9;17(3):e0265082. doi: 10.1371/journal.pone.0265082 (PMC8906646; doi:10.1371/journal.pone.0265082)
Supplement: S3 Table — (DOCX) [file pone.0265082.s004.docx]

**S3 Table. Estimates of the adjusted hazard ratios of variables on the development of delirium in the Cox-proportional hazards models removing patients with dementia or mental disorders from the primary analysis.**

| **Variable** | **Adjusted**  **hazard ratio** | **95% CI** | **p-value** |
| --- | --- | --- | --- |
| **No-visitation policy** | 0.890 | 0.572-1.385 | .607 |
| **Age** | 0.995 | 0.976-1.014 | .606 |
| **Male** | 1.339 | 0.802-2.235 | .262 |
| **Emergency surgery** | 1.352 | 0.590-3.098 | .475 |
| **APACHE II**^a^ | 1.038 | 1.011-1.067 | .005 |
| **Benzodiazepine use** | 1.419 | 0.747-2.694 | .284 |
| **Mechanical ventilation use** | 1.895 | 1.065-3.380 | .029 |

^a^APACHEⅡ score was calculated without age related score
